# Multifunctional Nanostructures with Controllable Band Gap Giving Highly Stable Infrared Emissivity for Smart Thermal Management

**DOI:** 10.1021/acsnano.2c09737

**Published:** 2023-01-09

**Authors:** Michal Delkowski, José Virgilio Anguita, Christopher Toby Gibb Smith, S. Ravi P. Silva

**Affiliations:** Advanced Technology Institute, Department of Electrical and Electronic Engineering, University of Surrey, Guildford, SurreyGU2 7XH, United Kingdom

**Keywords:** thermal control, multifunctional nanothin films, nanocomposite materials, functionalized materials, superlattice structure

## Abstract

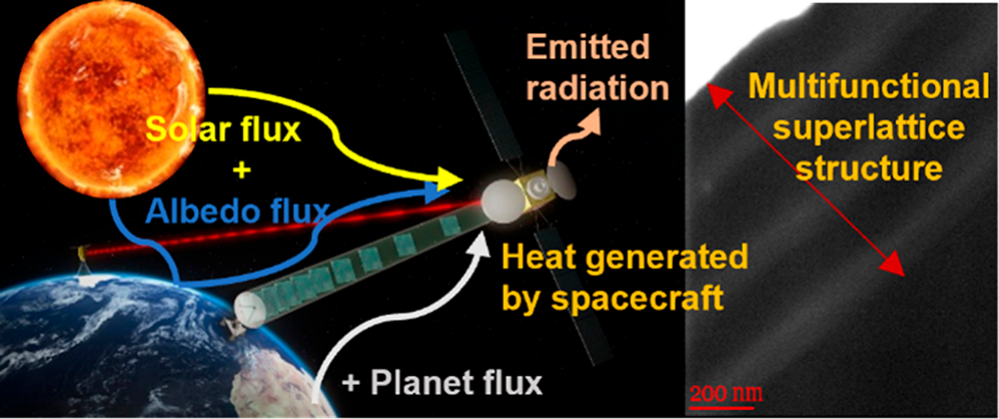

Thermal control is essential to guarantee the optimal performance of most advanced electronic devices or systems. In space, orbital satellites face the issues of high thermal gradients, heating, and different thermal loads mediated by differential illumination from the Sun. Todaýs state-of-the-art thermal control systems provide protection; however, they are bulky and restrict the mass and power budgets for payloads. Here, we develop a lightweight optical superlattice nanobarrier structure to provide a smart thermal control solution. The structure consists of a moisture and outgassing physical barrier (MOB) coupled with atomic oxygen (AO)–UV protection functionality. The nanobarrier exhibits transmission and reflection of light by controlling the optical gap of individual layers to enable high infrared emissivity and variable solar absorptivity (minimum Δα_S_ = 0.30) across other wavelengths. The multifunctional coating can be applied to heat-sensitive substrates by means of a bespoke room-temperature process. We demonstrate enhanced stability, energy-harvesting capability, and power savings by facilitating the radiation cooling and facility for active self-reconfiguration in orbit. In this way, the reduction of the operating temperature from ∼120 to ∼60 °C on space-qualified and nonmechanically controlled composite structures is also demonstrated.

## Introduction

**Figure 1:**
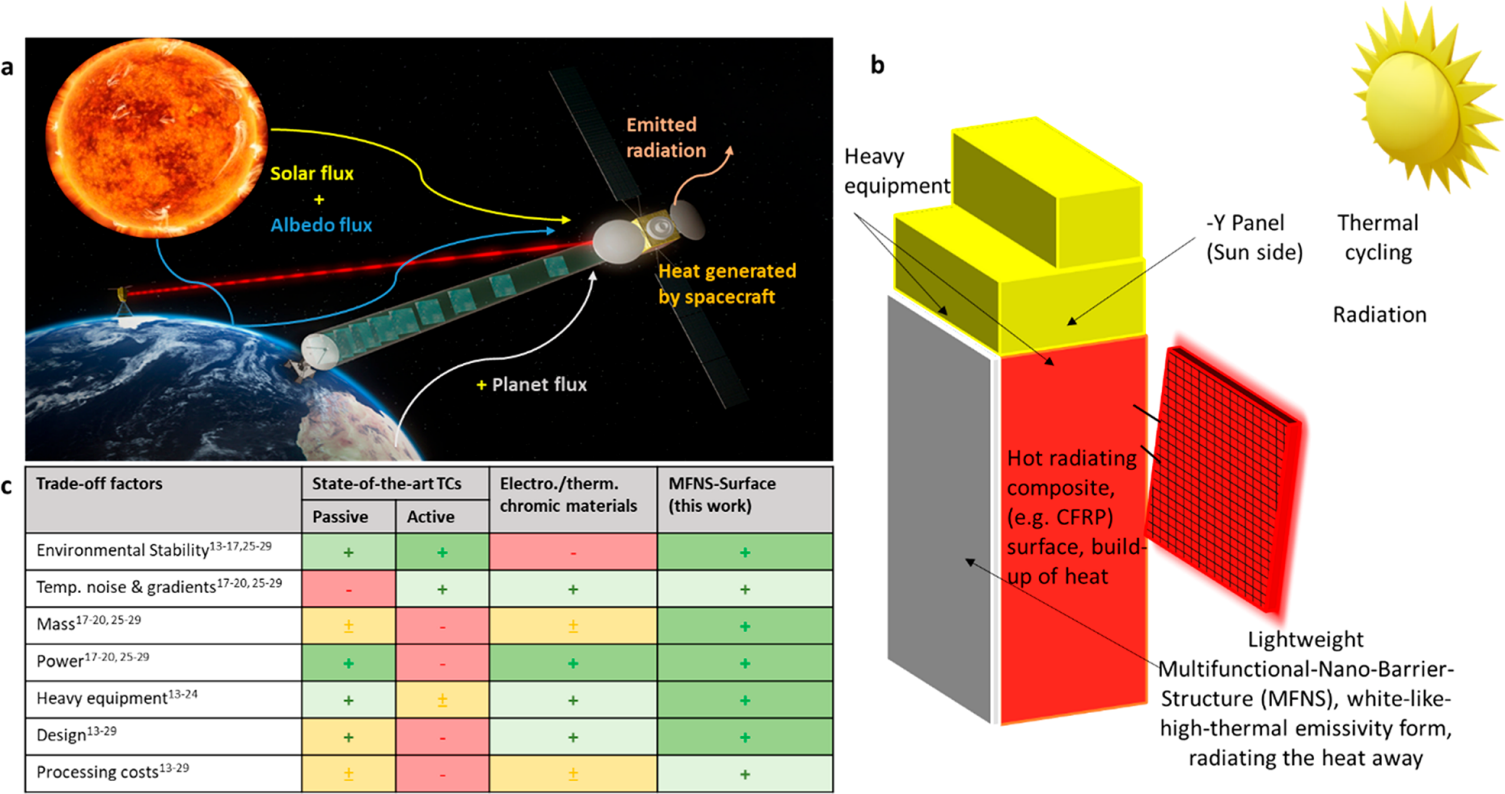
Challenges for orbiting satellites and TCSs. (a) Source of heat absorbed, stored, and dissipated by the spacecraft. (b) Schematic illustration of the satellite (Sun side) and TCSs, including a MFNS (this work) enhancement via white-like high-thermal emitting surface. (c) Simplified comparison of state-of-the-art TCSs (described in the text) to the developed MFNS (this work). Colors reflect the compliance with applications and/or adaptability: red, limited; yellow, moderate; light green, good; dark green, very good.

Thermal control is essential for spacecraft systems, in order to maintain the temperature of the spacecraft within a narrow range of operating temperatures during the operational lifetime.^1,2^ The design of thermal control systems (TCSs) on satellites is dictated by the extreme conditions of the harsh environment in space. Large temperature differentials across mission payloads can exert dimensional changes that result in mechanical stresses, which must be mitigated, to avoid structural distortions and ensure mission success.^3−8^ Without TCSs, these dimensional changes can result in misalignment of scientific instruments: i.e., optical components that are required to observe deep space and discover or monitor planets, as demonstrated recently.^9−12^ The spacecraft temperature is maintained by means of a delicate balance among external fluxes, emitted radiation, and heat that is produced internally (Figure 1a). State-of-the-art TCSs include passive methods such as thin foils, multilayer insulation (MLI), sun shields, etc. or active methods such as electrical heaters, cryo-coolers, etc., which require power for operation.^13−16^ Thermal radiators are used to dissipate heat away into deep space, while heating technologies such as heat pipes ensure minimal heating so that mission payloads can withstand the coldest boundary conditions, also known as the “cold case”. A combination of both active and passive control techniques is usually required for large satellites (Figure 1b) to ensure temperature control within very tight margins. However, their application is limited by the power and mass budgets, which often result in the cancellation of mission studies.^17−20^ Smaller satellites, such as CubeSats, require smaller and lighter thermal management solutions due to the size limitations, which restrict the utilization capacity of active systems^21−24^ (Figure 1c). A good example is the commonly used thermal louver developed by the National Aeronautics and Space Administration (NASA) that uses bimetallic springs to control the position of the flaps. Here, when the temperature of the spacecraft rises, the springs expand, opening the louvers to modify the average IR emissivity of the exterior surface or, when the spacecraft cools, the flaps close and the exterior surface returns to the previous optical properties. However, this mechanical solution requires additional equipment, mechanisms, mass, and input power and thereby is limited on small satellites. Also, sunshields such as that used for the James Webb Telescope (JWST) exert a high mass and complexity, as these are often-deployed devices. Furthermore, JWST and other sensitive instruments required additional heaters to maintain temperature, cleanliness, and structural performance of the instruments and satellites. This further added extra mass and complexity.^13−29^ Various smart electrochromic and thermochromic materials that are designed to operate over a selected range of wavelengths have also been investigated as a means of achieving variable thermal control, which would allow seamless operation. However, these materials usually require heavy equipment and they only vary the thermal emittance in the infrared spectrum (e.g., architectures based on vanadium dioxide (VO_2_) films with an emissivity change from 0.13 to 0.68).^25−27^ Furthermore, they are sensitive to thermal stresses and impacted by changes in levels of moisture.^25,28,29^ This has been shown and rejected for the Sentinel missions under the Copernicus Earth Observation Programme.^30−32^

This article presents a multifunctional nanobarrier structure (MFNS) that can be applied to satellite surfaces such as polymers, carbon fiber composite materials, MLI, photovoltaics, and photonics for adjustable and variable thermal and photonic spectrum management. The MFNS is a lightweight, multilayer protection nanobarrier, which is deposited via a custom-built plasma-enhanced chemical vapor deposition (PECVD) system without breaking vacuum, where the deposition takes place at room temperature. The MFNS can be modulated to provide adjustable solar absorptivity (α_S_) in the UV–visible range, while simultaneously enabling high and stable infrared (IR) emissivity. This can be reconfigured in orbit by means of balancing the UV and atomic oxygen (AO) exposure. Furthermore, a selective high-emissivity structure can be realized to enable energy harvesting via a photothermal energy conversion efficiency with limits as high as 96.66%.^30,31^ Finally, it is shown that the MFNS can be used along with embedded sensors and advanced composite materials for sensing and property transfer, resulting in smart thermal management on radiators and satellite surfaces. Consequently, this technology enables many smart technologies based on photonics, electronics, and photovoltaics in a wider field of applications.

## Results and Discussion

**Figure 2:**
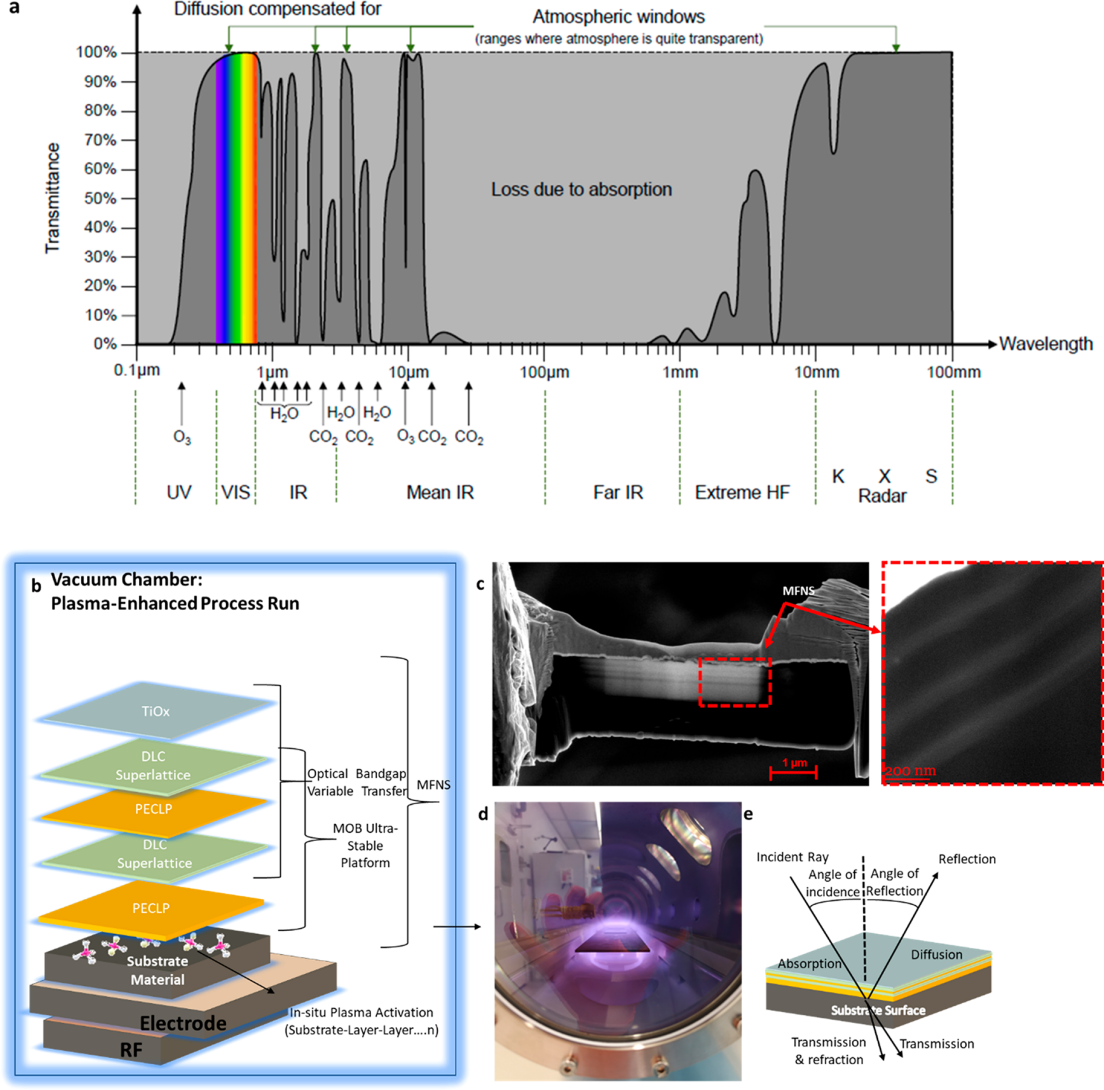
Electromagnetic radiation from space and custom-designed PECVD and MFNS. (a) Image showing electromagnetic radiation from space reaching Earth’s surface, considering effects of the atmosphere. (b) MFNS structure composed of a moisture and outgassing barrier (MOB) platform and TiOx transfer layer realized in one chamber without breaking vacuum at room temperature. (c) Scanning electron microscope (SEM) cross-section images showing a defect-free MFNS (parallel lines) deposited on a planar composite substrate, Scale bars: 1 μm and 200 nm. (d) Images showing a composite panel with glowing plasma during the MFNS deposition. (e) MFNS-coated substrate with depicted optical effects for the MFNS–substrate system.

One approach to overcome the high power/mass budgets typically required by thermal control systems of satellites is to provide a selective adjustment of both solar absorptivity (α_S_) and infrared emissivity (ε_IR_) smart coatings in order to facilitate radiative cooling, while harvesting the electromagnetic radiation in space (Figure 2a). It has been shown that, with this approach, it is possible to achieve power savings nearing 100%.^32^ However, designing a material that actively adjusts α_S_ and ε_IR_ according to spacecraft requirements is challenging.^25−27^ Thin-film optical structures are often vulnerable to degradation or delamination on exposure to the harsh environment of space. These failure modes result from a mismatch of the coefficient of thermal expansion (CTE) between the film and substrate.^33−35^ Furthermore, these structures are usually delicate and difficult to handle and deteriorate when manually handled.^32−41^ The CTE mismatch is especially exacerbated for carbon-fiber polymer (CFRP), a material that is often a choice for fabricating platforms, support structures, optical benches, telescope tubes, and parabolic reflectors among other satellite components due to its strength and light weight. Here a MFNS (Figure 2b) that consists of a plasma-deposited poly(*p*-xylylene) (PECLP) buffer layer and high-density diamondlike-carbon (DLC) superlattice is used to create a mechanically and environmentally ultrastable platform.^36,37^ The topmost layer of the ultrastable platform is a variable-oxide-controlled TiOx heterostructure layer that is deposited to enhance the protection against degradation from atomic oxygen (AO) and UV radiation. The combined layer is a dielectric and is therefore electromagnetically transparent across a wide range of radio frequencies. It can therefore be used to coat antennae structures without adding significant interference to the signal.^38^

A custom-built plasma-enhanced chemical vapor deposition (PECVD) system is used for coating, where the substrates are coupled to the radio frequency (RF) plasma (13.56 MHz), creating a virtual electrode. This allows the complete deposition of MFNS by direct coupling to the electrical power supply via electromagnetic (EM) fields. To ensure treatment of heat-sensitive or rough substrates such as polymers, a room-temperature conformal coating process was used. The PECLP layers deposited exhibit increased robustness and elastic modulus and an intermediate CTE of ∼35 ppm.^37^ The DLC superlattice is formed using hydrocarbon gas sources in a PECVD process. The barrier layers were constructed from a dense and sp^3^-rich (sp^2^/sp^3^ ratio ∼8%) DLC, as measured by electron energy loss spectroscopy (EELS) with a transmission electron microscope (TEM).^38^ Furthermore, the DLC superlattice is used as a moisture barrier and as a barrier to volatile condensable materials (CVCM = 0.000%) from outgassing, as shown by microvolatile condensable material (μ-VCM) measurements. These water and outgassing effects typically degrade the photothermal characteristics of materials and devices.^39−41^ TiOx layers were deposited from vapor sources of titanium isopropoxide (TIPPs). The oxygen content of these was determined using X-ray photoelectron spectroscopy (XPS) and energy-dispersive X-ray spectroscopy (EDX).

**Figure 3:**
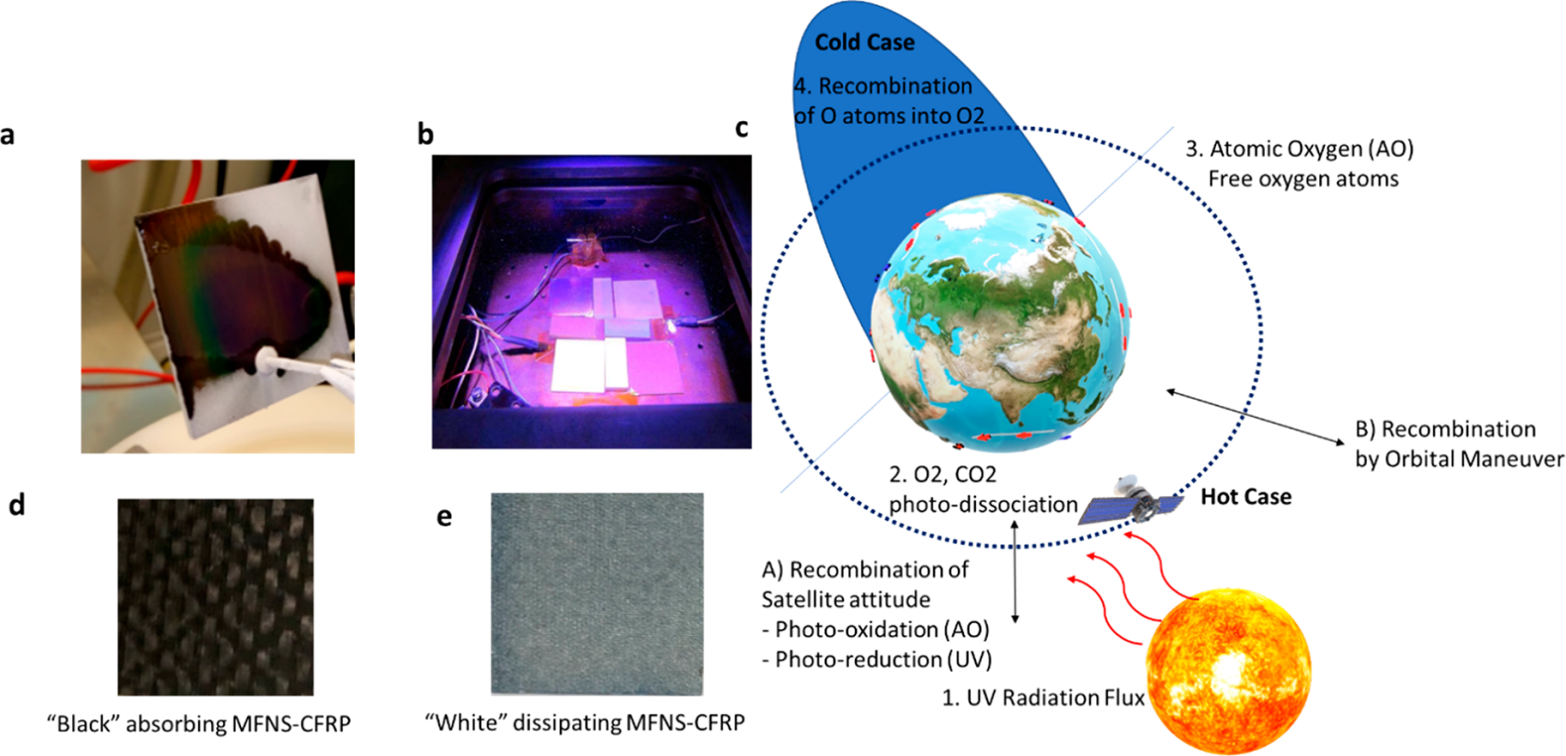
Thermo-optical design and testing of MFNS. (a) MFNS-coated CFRP after undergoing thermal cycles at ∼−200 °C in liquid nitrogen: e.g. during sun eclipse, space exploration. (b) MFNS-coated polymeric and composite materials during space-simulated UV irradiation tests. (c) Schematic illustration showing an orbiting satellite with “hot and cold cases” and explanation of mechanisms 1–4 for UV irradiation, atomic oxygen creation via photodissociation as well as its recombination resulting in MFNS reconfiguration by adjusting satellite orientation, attitude and/or orbit (A, B). (d) MFNS with designed narrow optical band gap (UV–vis–IR) enabling a black-body structure for stray-light and thermal control in optical applications. (e) MFNS with designed wide optical band gap (UV–vis) enabling a white-body structure for enhanced heat dissipation.

A typical orbit of a satellite around Earth is divided into two phases: (1) Sun-lit phase and (2) eclipse phase (Figure 3). The duration of these phases depends on the type of orbit and altitude, each resulting in specific hot and cold environments that are addressed and tested in this work. A typical range of temperature is from ∼−175 to +125 °C for low-Earth-orbit (LEO) satellites, while a temperature range from ∼−250 to +300 °C is experienced in other orbits.^14−19^ This temperature range can include other effects such as an aero-braking phase, which is planned for the approach to Venus by NASA and other space agencies.

Consequently, to demonstrate the MFNS capability to withstand the harsh temperature extrema required for space travel, MFNS was subjected to thermal cycles from ∼−200 to +300 °C. The samples exhibited mechanical integrity to these extreme thermal cycles. This temperature range is consistent with external satellite surface temperatures, thereby demonstrating the thermomechanical integrity of MFNS during simulated spaceflight. This included the humidity (85% RH, 60 °C) exposures and validation of coating adhesion, which was confirmed using tape tests (e.g. Figure S5) before and after thermal cycling and after other synergistic space conditions such as atomic oxygen (AO) and UV irradiation, which are subsequently discussed.

To characterize the optical properties of the MFNS, an optical band gap analysis was carried out using spectroscopy, alongside with the determination of the optical band gaps using the Tauc relationship
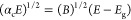
1where *α*_c_, *E*, *E*_g_, and *B* are the absorption coefficient (in cm^–1^), photon energy *h*ν, Tauc band gap, and a constant relating to the Urbach energy tail, respectively. The optical band gaps for each layer were in the ranges 1.5–2.8, 1.5–4.0, and 3.0–3.6 eV for PECLP, DLC superlattice, and TiOx heterostructure coating, respectively.^36−38,42^ In order to enable the radiation cooling on antenna or microwave surfaces, the MFNS was engineered to have a higher reflectivity in the UV–vis range to achieve a lower value for solar absorptivity (α_S_) and simultaneously high and stable IR emissivity (ε_IR_).

**Figure 4:**
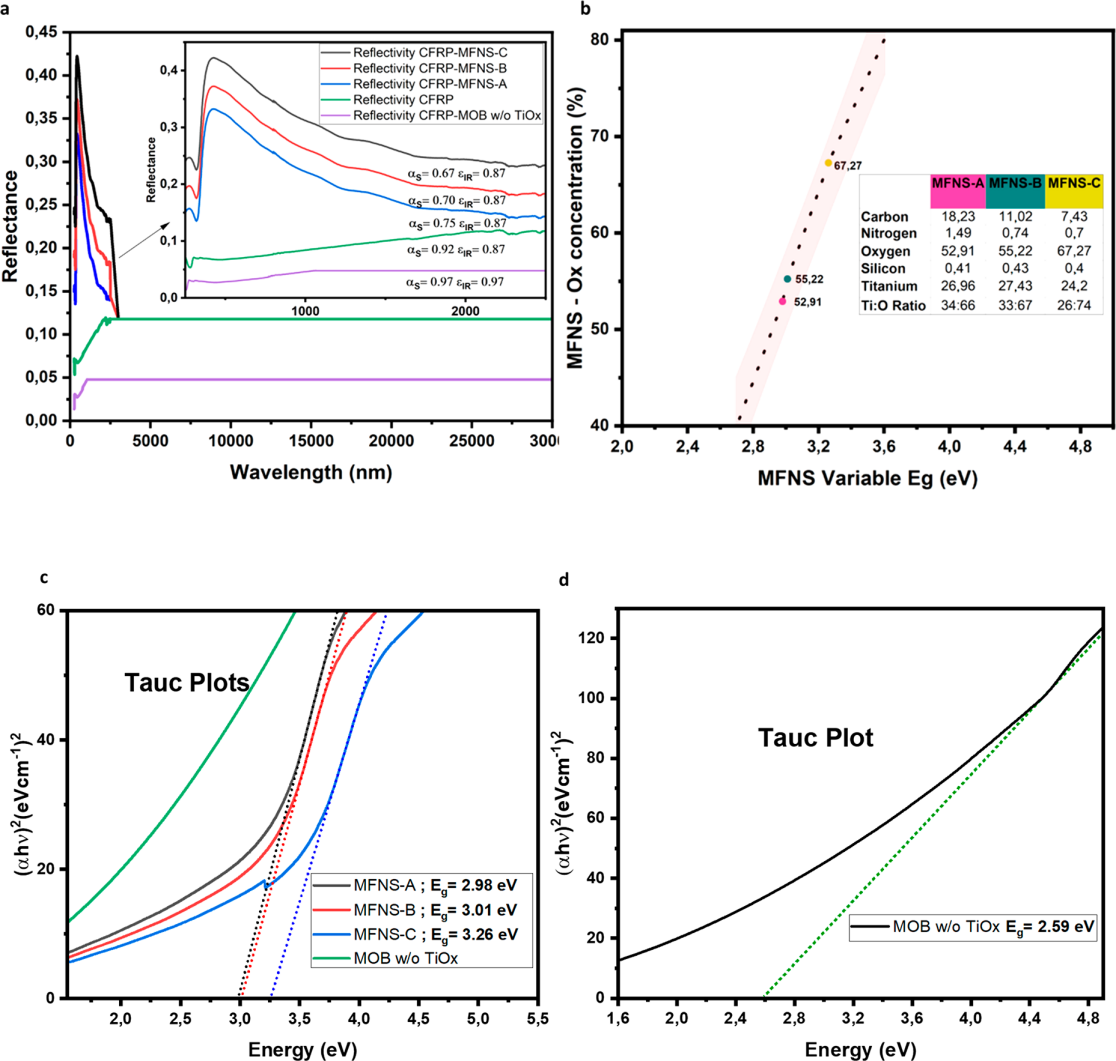
Thermo-optical characterization of MFNS. (a) MFNS with and without TiOx (i.e., merely MOB)-coated CFRPs with modulated reflection showing differential solar absorptivity (α_S_) with a stable and high IR emissivity (ε_IR_) for better thermal dissipation, as well as with high α_S_ and high ε_IR_ for an enhanced photothermal effect using a black-body-absorbing structure. (b) MFNS with variable band gaps in relation to the oxygen concentration and Ti:O ratio (MFNS-A, MFNS-B, MFNS-C), with a table showing the elemental composition of the surfaces of MFNSs determined using XPS. (c) Tauc plots for MFNSs with different oxygen concentrations. (d) Tauc plot for MOB with a narrowed band gap for a black-body-absorbing structure.

Figure 4a shows the thermo-optical characteristics of pristine CFRP (α_S_ = 0.92, ε_IR_ = 0.87), which is commonly used in satellites and advanced applications^36,3−6^ (Figure 1b). However, it is vulnerable to degradation when it is externally exposed to the space environment.^36−38^ Also, its thermo-optical properties cause the buildup of heat. However, by depositing the MFNS, which features a wide band gap, it is possible to achieve a white-like reflecting surface on CFRP (Figure 3e) with α_S_ and ε_IR_ values equal to 0.67 and 0.87, respectively. This is shown in Figure 4a. Here, the highest excess of oxygen atoms in the MFNS (Figure 4b,c) opens the band gap of TiOx to values greater than 3 eV, resulting in improved photothermal performance. The PECLP and DLC superlattice feature high IR emissivity across a wide spectral range (Figure 4a, showing a spectrum up to 30 μm). With this, the MFNS-C shows an optical band gap of 3.26 eV. Figure 4a shows that the MFNS is capable of changing the absorption in the UV–vis range with minimum Δα_S_ = 0.30, by balancing the Ti:O atomic ratio. Here, the oxygen concentration is modulated between 52 and 67% across MFNS types. For MFNS-A this results in a band gap of 2.98 eV, with a Ti:O ratio of 34:66. MFNS-B has a band gap of 3.01 eV, with Ti:O ratio of 33:67, and MFNS-C has a band gap of 3.26 eV, with a Ti:O ratio of 26:74, respectively (Figure 4b,c). This UV–vis absorption range, and thus the MFNS optical band gap, can be further widened to 3.4–3.6 eV by increasing the oxygen concentration and thus the Ti:O ratio to approximately 20:80 (Figure 4b).

**Figure 5:**
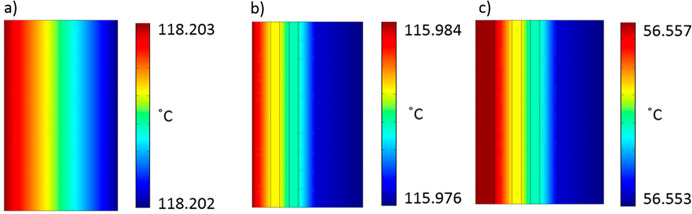
Thermo-optical computer model of uncoated CFRP compared to MFNS + CFRP. (a) Uncoated, standard CFRP used for satellite structures. CFRP coated with MFNS (b) without TiOx and (c) with TiOx. In all cases, the structures were modeled at thermal equilibrium in space, where one side is facing the Sun and the rear side is facing deep space. The different layers of the MFNS can be seen in the models.

Thermo-optical computer modeling of these structures in thermal equilibrium during spaceflight is shown in Figure 5. This shows the significance of emissivity coatings for managing heat in spaceflight. Figure 5a shows a section of the top surface of CFRP (uncoated) that is being exposed to sunlight on the front side and deep space on the rear side. The model shows the temperature variation close to the surface that is exposed to the Sun, about 1 μm deep into the surface. The model suggests an equilibrium temperature of around 118 °C at the Sun side. Using the MOB structure and the same optical constants as for uncoated CFRP (Figure 5b) results in a steeper thermal gradient (∼70 °C cm^–1^) and hence concentration of heat toward the top surface of the CFRP, which results from the use of PECLP and DLC on the front surface. These materials feature a low thermal conductivity of the stack and thus reduce the amount of heat flow to the deeper parts of the MOB and CFRP. Although the layer is thin, this results in a temperature savings of more than 2 °C. Using a white TiO_2_ reflective coating (Figure 5c and MFNS structures) results in a significant proportion of the radiation reflected away from the top surface and thus a significant reduction of equilibrium temperature to ∼56.5 °C (∼60 °C lower temperature than uncoated CFRP). This reduction of temperature is exacerbated by the trapping of heat between the TiO_2_ and the CFRP by the PECLP and DLC layers, which are directly beneath the TiO_2_. This acts as a significant barrier to heat propagation to the bulk of the CFRP and the delicate structures in the payload of the satellite.

The temperature gradient in the coated model (Figure 5c) shows that the temperature gradient across the hottest parts of the MFNS is ∼30 °C/cm, which is less than half that of the case without the TiO_2_ reflective coating. This translates to a significant lowering of thermal load and also significant reductions in mechanical stress to the structure.

**Figure 6:**
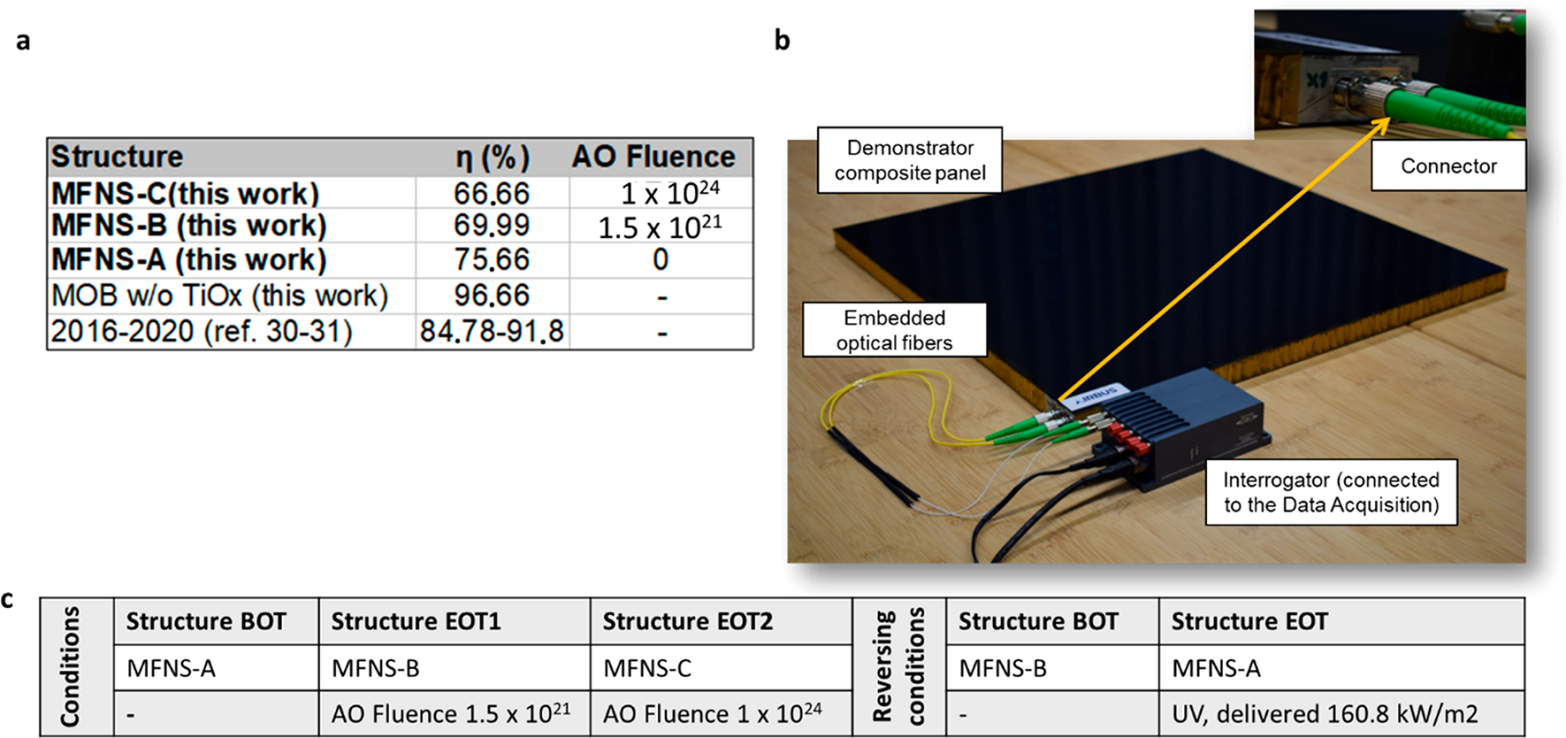
Demonstration of both variable and enhanced total photothermal conversion efficiency via MFNS as well as an intelligent nerve system for sensing and data/power transfer. (a) Measured photothermal energy efficiency on CFRP face-sheet panels with possible self-reconfiguration in orbit between MFNS-A and MFNS-B under AO fluence and a table reporting a photothermal energy conversion efficiency on CFRP face-sheet panels enhanced by various MFNSs. (b) Composite system (approximately 1 m × 1 m) with embedded optical fibers and fiber Bragg gratings (FBGs) for thermomechanical environmental control and signal/power transfer. Inset: a plug-and-play solution for embedded optical fibers. (c) Table summarizing reverse effects between MFŃs when balancing UV and AO exposure (BOT, beginning of test; EOT, end of test).

To simulate the irradiation effects from the harsh environment in space, UV irradiation tests were carried out (Figure 3c). Uncoated cross-linked polystyrene materials which are commonly used for microwave applications show a change in solar absorptivity and IR emissivity values, which result in a worsening radio frequency (RF) transparency performance (Figure S1).^38^ The relative permittivity deteriorated from 2.467 to 2.228, resulting in higher RF losses. In contrast, MFNS-protected substrates showed stable photothermal characteristics (before and after the test), thereby ensuring stable RF properties (relative permittivity 2.477 before and after the test). A primary source of degradation in space is from exposure to atomic oxygen (AO), which is created by dissociation of molecular oxygen in the upper atmosphere by UV radiation, to form AO radicals commonly found in space, around LEO (Figure 3).^1,38^ To replicate AO conditions on Earth, MFNS samples were first exposed to a cumulated average fluence of 1.1 × 10^21^ atoms/cm^2^ (further extended to 1 × 10^24^ atoms/cm^2^ for MFNS-C) in order to simulate a satellite mission lifetime of several years. The average AO kinetic energy was 5 eV, resulting in MFNS-A transforming into MFNS-B by end of the test and then to MFNS-C with extended exposure. This is shown in Figure 6a,c, where, with excess oxygen, the solar-thermal conversion efficiency is increased and thereby the absorption is decreased to 69.9% for MFNS-B (1.1 × 10^21^ atoms/cm^2^ AO) and to 66.66% for MFNS-C (1 × 10^24^ atoms/cm^2^ AO), whereas with the lower oxygen content (MFNS-A) a value of 75.66% was achieved.

As the dissociation of molecular oxygen is realized via UV light (Figure 3), this inspired the use of UV irradiation itself to revert the MFNS-B to MFNS-A (Figure 6c). This was achieved upon delivering 160.8 kW/m^2^ at an increased temperature of 250 °C to accelerate conditions, which corresponded to 1227 equiv Sun hours (ESH) with a solar spectrum (AMO) power of 131 kW/m^2^ between 200 and 415 nm. This showed the capability of MFNS for smart control of the temperature, which is achieved by adjusting the satellite orientation, attitude, or orbit (Figures 1 and 3 showing recombination mechanisms considering a sun side or nadir-pointing mode).

With respect to the high-absorbing structures which are typically required for stray-light control in optical instrumentation, or thermal management in thermophotovoltaics devices, a black-body MFNS (MOB itself) with a narrow band gap was designed (Figures 3d and 4d). Both α_S_ and ε_IR_ values of ∼0.97 were obtained across the UV–vis–IR range, as measured according to the ECSS-Q-ST-70-09C standard. This was possible by the deposition of a nitrogen-doped DLC superlattice layer (DLC:N) which features a narrow band gap (total MOB 2.59 eV, Figure 4d). DLC:N is a material that allows direct optical transitions and increased donor states within the band gap, thereby giving rise to enhanced optical absorption across a wide spectral range. The total photothermal conversion efficiency for various MFNSs is shown in the inset table (Figure 6a), where 96.66% of efficiency for the black-body MOB was achieved. This high level of absorption was stable throughout the entire UV irradiation exposure (cumulated power) during spaceflight simulation tests. This demonstrates a capability for both a high and adjustable photothermal effect for energy harvesting that may be realized by combining with intelligent embedded functions.

This approach is shown in Figure 6b, which shows a manufactured CFRP sandwich demonstrator with embedded optical fibers (Figure S2). This embedding together with the MFNS can be used for intelligent nerve sensing and data transfer functionalities where additionally eight fiber Bragg gratings (FBGs) optical strain sensors were inscribed onto the core of the optical fiber. A continuous measurement of thermal-cycle effects between −20 and +40 °C using these embedded optical fibers with FBGs is demonstrated (Figure S4a and Figure S3 showing measurement setup). This is used along with the ultrahigh-modulus (UHM) conductive carbon fibers to enable enhanced photothermal coupling among the MFNS, MOB, and composite substrates.^43,44^ These fibers have up to 99.9% carbon content (Figure S4b) and exhibit up to 800 W/mK thermal conductivity and less than 1.0 μΩ m electrical resistivity. These together with the developed MFNS (or MFNS alone) can enable a wide range of applications among photonics, electronics, photovoltaics, structural engineering, and water management. These enhanced properties, along with advanced manufacturing methods, demonstrate that the MFNS can be a candidate for many thermal applications such as photodetectors, emitters, smart radiators, and energy harvesting used in satellite systems and beyond.

## Conclusions

A technology is presented for the design and manufacture of an advanced multifunctional nanobarrier structure (MFNS) that features an adjustable photothermal response across a wide spectral range. This mechanically and environmentally robust multilayer barrier coating can control its optical band gap. This control is made possible by engineering a thermomechanically coupled multilayer structure on the substrate surfaces with embedded photothermal conversion effects by balancing the Ti:O ratio and modulating the DLC superlattice structure. This demonstrates capabilities to generate variable solar absorptivity (minimum Δα_S_ = 0.30) and stable IR emissivity characteristics with possible self-reconfiguration in a LEO when balancing AO/UV conditions and possible further passing to higher orbitals. A significant reduction of equilibrium temperature from ∼120 to ∼60 °C is demonstrated when using the highly reflective MFNS on the CFRP. The MFNS is light weight (submicrometers thick) and enables these without requiring external power inputs, which shows significant improvement over the conventional space-qualified TCSs, thereby allowing advanced architectural concepts with respect to the mass/power ratio on satellites. A range of materials can be used, utilizing the MFNS as a stable platform to adjust further properties. Furthermore, highly absorbing structures can be achieved for energy harvesting with a photothermal conversion efficiency as high as 96.66%. This technology can be combined with embedded optical fibers into the materials, to form a range of smart devices, including highly conductive reinforcements for composites, radiation coupling by heat spreads, additive manufacturing, or embedded micropipe networks for generating smart devices with property transfer (Figure 6b) for structural and environmental sensing as well as thermomechanical and optical adjustments.

## Materials and Methods

A MFNS was deposited using a custom-built PECVD coating system at room temperature where substrates were directly connected to the electrically driven electrode, thereby creating a virtual electrode (Figure 2b). The buffer layer was PECLP, the hard inorganic barrier layers were plasma-deposited DLC, and the top layer was TiOx. The initial PECLP layer was typically 500 nm thick, and the first DLC layer was 50, 100 or 200 nm thick. The second and subsequent PECLP coating layer(s) that was deposited on the top of the initial PECLP:DLC superlattice was 100 nm thick. The MOB coating was deposited to mitigate surface stresses of the substrates and increase the adhesion as well as provide a moisture and outgassing barrier. The TiOx layer was 200 nm thick. To deposit PECLP, a dimer di-para-xylylene was used, where further monomer was introduced into the plasma within the PECVD chamber. A hydrogen/acetylene mixture was used to deposit the DLC superlattice where hydrogen acted as a carrier gas, carrying the acetylene precursor. Titanium isopropoxide (TIPP) vapor was introduced through heated pipework into the PECVD chamber for TiOx deposition. The Ti:O ratio was balanced by plasma oxidation processing. Additionally, transforming between various MFNSs (A–B–C) was achieved after atomic oxygen and UV irradiation exposures.

μVCM measurements were performed according to the Space Product Assurance document (no. ECSS-Q-ST-70-02C: European Cooperation for Space Standardization of space activities) in a dedicated European Space Agency (ESA) laboratory in Madrid. These were performed under a high vacuum at 65 and 125 °C, while the total amount of collected volatile condensable materials (CVCM), such as water and volatile organics, was collected using a microbalance plate cooled to ∼−200 °C using liquid nitrogen for analysis using infrared spectroscopy. Thermal cycling in the ranges −60 to +80 °C and −20 to +40 °C were performed in a thermal vacuum chamber with a typical heating rate of 10 °C/min. Additionally, thermal cycling tests were performed several times during development by exposing substrates to up to 100 cycles ranging from ∼−200 to +300 °C. Adhesion tape tests were carried out on all MFNS-coated substrates before and after particular tests to verify the coating robustness (e.g., Figure S5). At least 5 cm of pressure-sensitive tape was attached on the surface with pressure applied to ensure it adhered well to the surface. The tape was then pulled at right-angles at a rate of approximately 1 cm s^–1^. A remnant of coating was inspected on the tape, if any. All components were tested and had to pass an adhesion tape test after undergoing thermal cycling in liquid nitrogen.

A Dektak profilometer, scanning electron microscope (SEM, using FEI Quanta), transmission electron microscope (TEM), and *in situ* quartz oscillator measurements were used to inspect and measure the MFNS thickness. Optical measurements were taken using a Cary 5000 UV–visible–IR light spectrophotometer up to 2500 nm to cover a broad spectrum. Additionally, a portable emissometer/reflectometer (TESA 200b) was used to perform optically integrated total hemispheric reflectance measurements from less than 3 μm to greater than 35 μm wavelength. The measurements were performed according to the ECSS-Q-70-09C standard (measurements of thermo-optical properties of thermal control materials). The XPS, EDX, Raman, and EELS/TEM measurements were used for each layer and MFNS (Figure 4).

Modeling was performed using COMSOL Multiphysics, using the heat transfer module for heat transfer, convection, and radiation. The thermal properties of the composite with the thermo-optical stack during spaceflight were modeled using the Stefan–Boltzmann relation and the emissivity of the various surfaces.

The LEOX atomic simulator at the European Space Research and Technology Centre (ESTEC) was used to simulate atomic oxygen, where oxygen gas was detonated using a CO_2_ pulsed laser and accelerated to approximately 8 km/s to represent conditions experienced in a low Earth orbit. The average AO kinetic energy was 5 eV, and Kapton witness samples were used to enable a fluence map control. UV irradiation was performed using a Newport Oriel solar simulator with a xenon arc lamp as a light source. The device emits a 5800 K black-body-like spectrum with an occasional line structure. The UV range was between 200 and 415 nm with light intensity averaging ∼660 W/m^2^ and reaching an equivalent of 5 Sun exposure intensity. 1227 equivalent Sun hours (ESH) were reached, while the working distance of the light source was adjusted to ensure the temperature of samples at 60 °C, further increased to 250 °C to accelerate, and thus observe reversible behavior.

A split post dielectric resonator (SPDR) from QWED operating at 10 GHz was used to determine the complex permittivity of materials. In addition, a WR90 waveguide cavity resonator was used to measure samples over a broad-band range at 10 GHz. A vector network analyzer (NWA) was used to measure the amplitude and the phase of scattering parameters on the polymeric materials (dielectric constant and loss factor). This was for adjustment (return loss) between 5 and 13 GHz with 1601 frequency points. The patterns were measured between 7.5 and 12.5 GHz with 101 frequency points.

A composite demonstrator with embedded optical fibers and FBGs was manufactured at Airbus Defense and Space GmbH, Friedrichshafen. The layout is shown in Figure S2 with eight FBGs and connector included. Ultrahigh-modulus (UHM) fibers with an epoxy matrix system were used.

## Supporting Information Available

Thermo-optical characterization, CFRP–aluminum sandwich schematic, thermal-cycling data, continuous thermal sensing data, and thermal environment adhesion tape testing (PDF)
