# Supplementary material for: Multifunctional Nanostructures with Controllable Band Gap Giving Highly Stable Infrared Emissivity for Smart Thermal Management
Source: ACS Nano. 2023 Jan 9;17(2):1335–43. doi: 10.1021/acsnano.2c09737 (PMC9878728; doi:10.1021/acsnano.2c09737)
Supplement: Supplementary file 1 — nn2c09737_si_001.pdf [file nn2c09737_si_001.pdf]

Supporting Information

Multifunctional nano-structures with controllable bandgap giving highly stable infrared emissivity for smart thermal management

*Michal Delkowski, José Virgilio Anguita, Christopher Toby Gibb Smith and S. Ravi P. Silva**

Advanced Technology Institute, Department of Electrical and Electronic Engineering, University of Surrey, Guildford, Surrey GU2 7XH, United Kingdom.

Keywords: thermal-control, multifunctional nano-thin films, nano-composite materials, functionalized materials, superlattice structure.

AUTHOR INFORMATION

Corresponding Author

Prof. S. Ravi. P. Silva – Advanced Technology Institute, University of Surrey, Guildford, Surrey, GU2 7XH, United Kingdom – s.silva@surrey.ac.uk

ASSOCIATED CONTENT

Figure S1 - Thermo-optical characterisation at the beginning of AO/UV test (BOT) and end of AO/UV test (EOT).

Figure S2 - CFRP-Aluminium sandwich with embedded optical fibres and FBGs.

Figure S3 - Demonstrator CFRP/Aluminium sandwich panel during thermal-cycling test in a chamber.

Figure S4 - Continuous thermal sensing and highly conductive carbon fibres.

Figure S5- MFNS-CFRP before and after thermal environment adhesion tape testing.

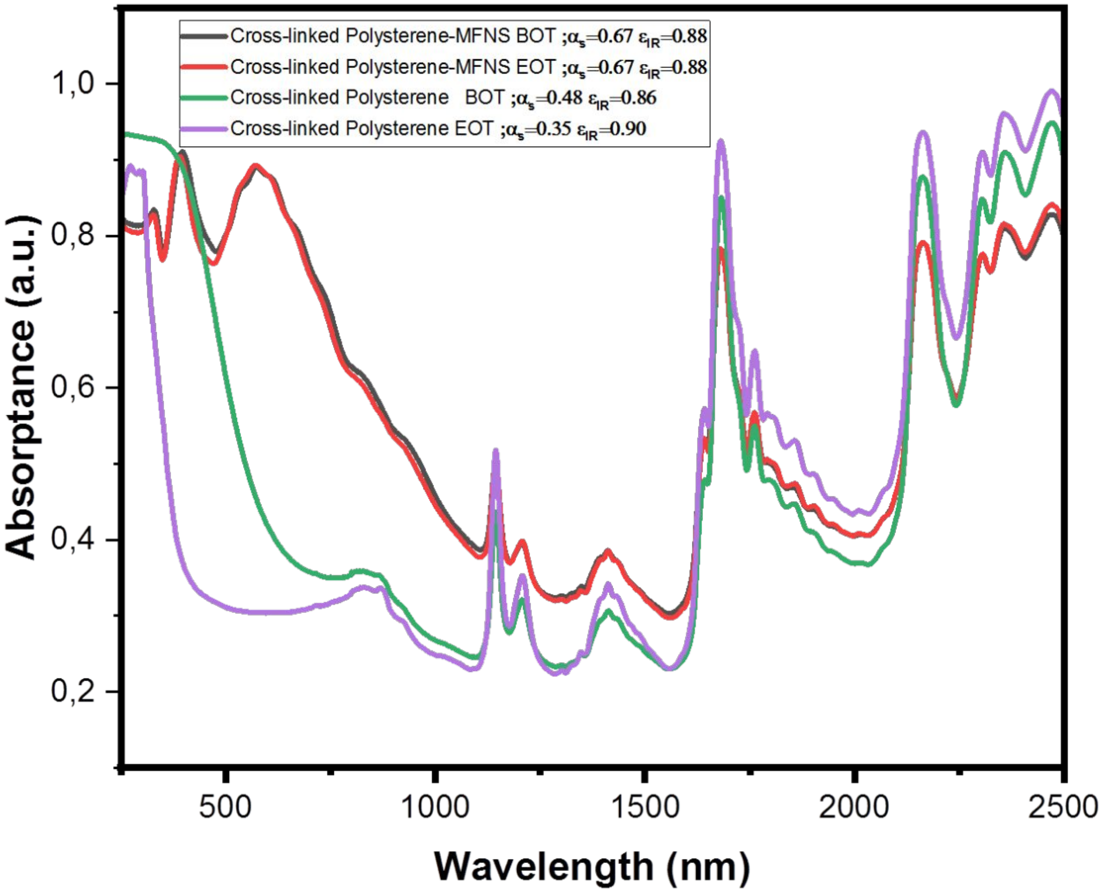

Figure S1: Thermo-optical characterisation at the beginning of AO/UV test (BOT) and end of AO/UV test (EOT) – MFNS cross-linked polystyrene showing stable behaviour (BOT=EOT) with UV protection by enhanced absorption in UV-VIS spectral range. Pristine cross-linked polystyrene showing photo-chemical degradation in UV-VIS spectrum which is consistent with a mass and microscopy characterisation.

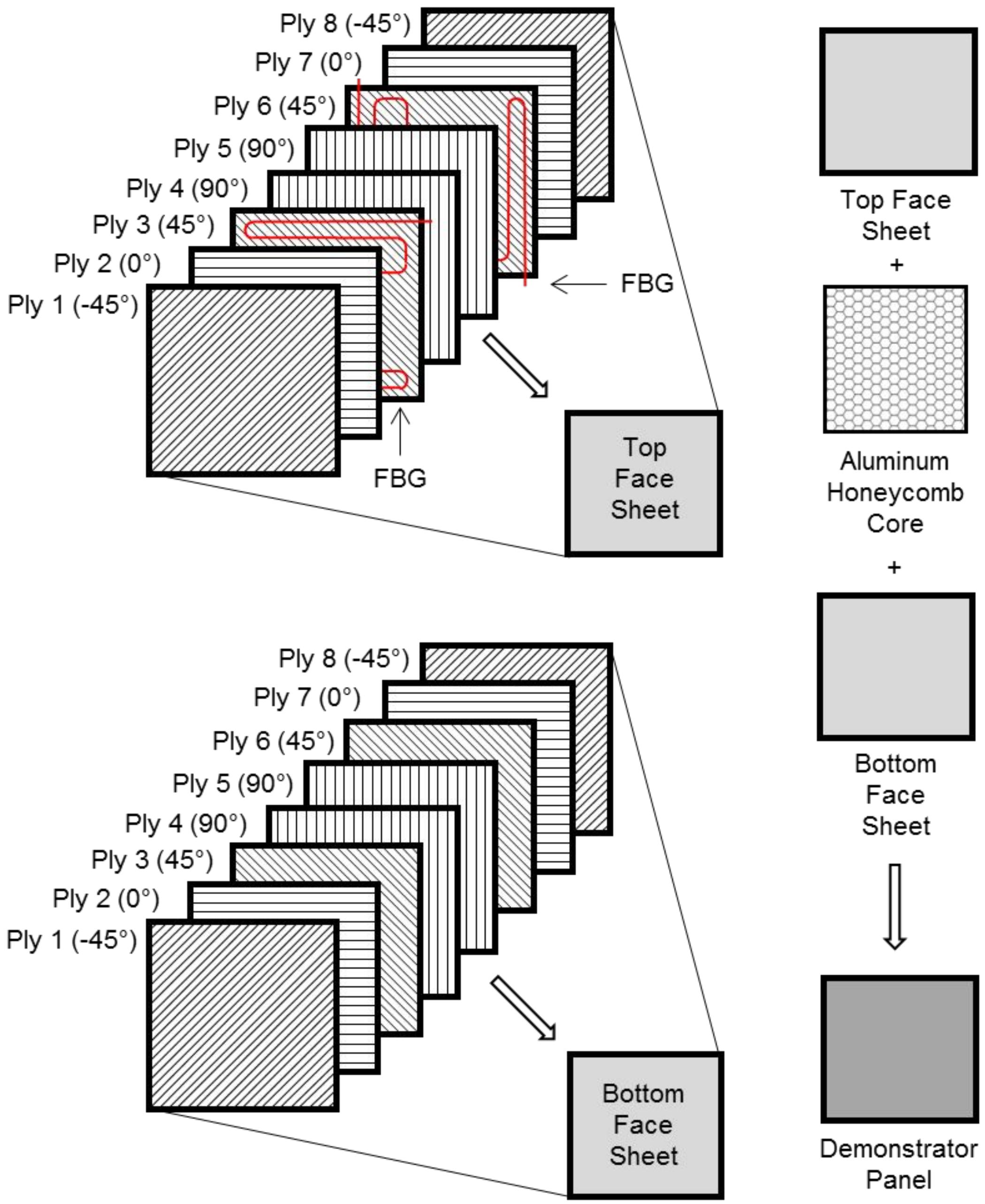

Figure S2: CFRP-Aluminium sandwich with embedded optical fibres and FBGs – Quasi-isotropic lay with optical fibres embedded into the top CFRP face-sheet (Ply 3 45˚ and Ply 6 45˚) with constructed sandwich (CFRP face-sheets + aluminium honeycomb core).

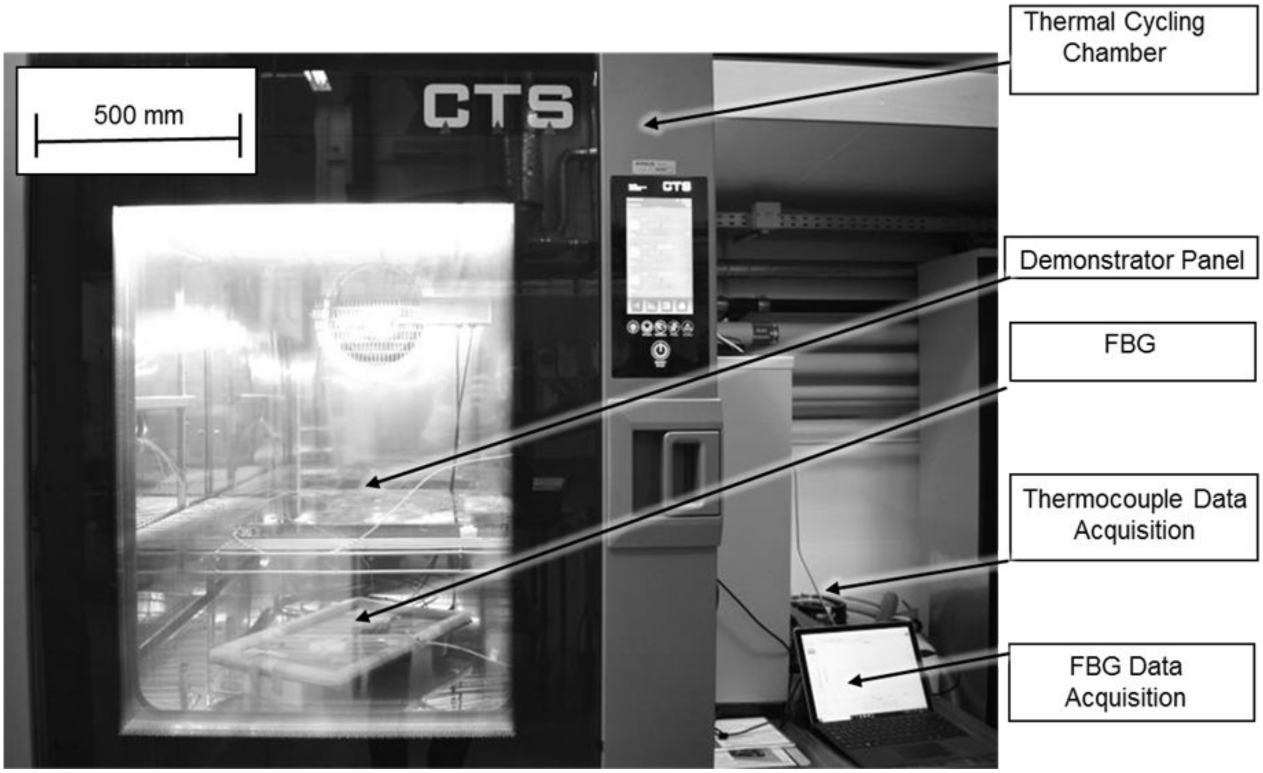

Figure S3: Demonstrator CFRP/Aluminium sandwich panel during thermal-cycling test in a chamber – Demonstrator panel (Figure S2) with a data acquisition system during testing.

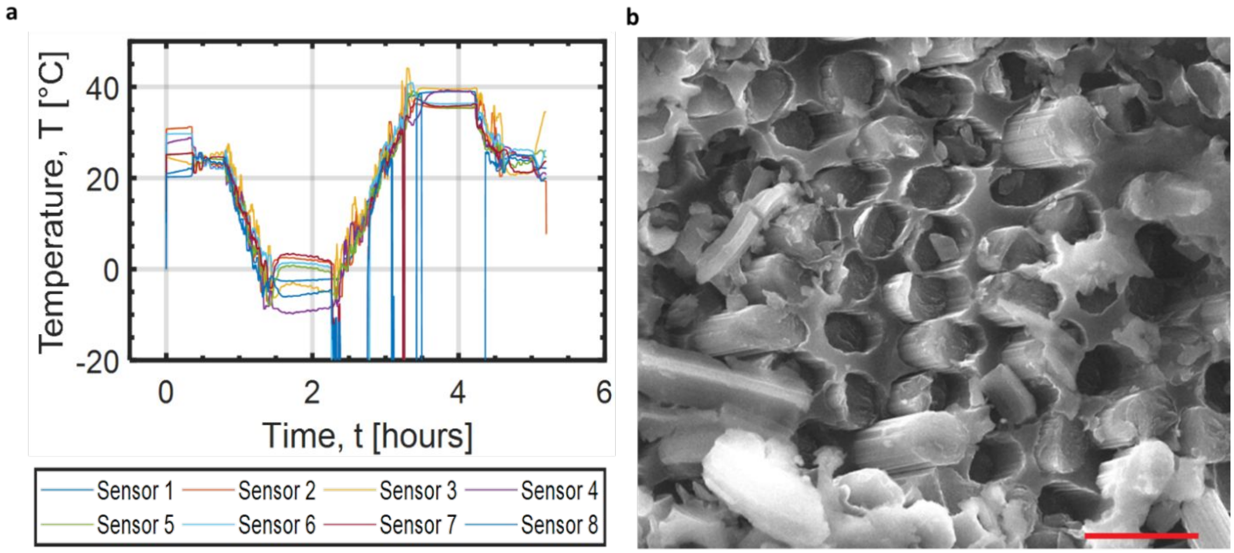

Figure S4: Continuous thermal sensing and highly conductive carbon fibres. a) Measured thermal cycling using embedded optical fibres with FBGs into the demonstrator (Figure S2 and S3), b) SEM image showing highly conductive PITCH carbon fibres in a CFRP (scale bare, 10μm).

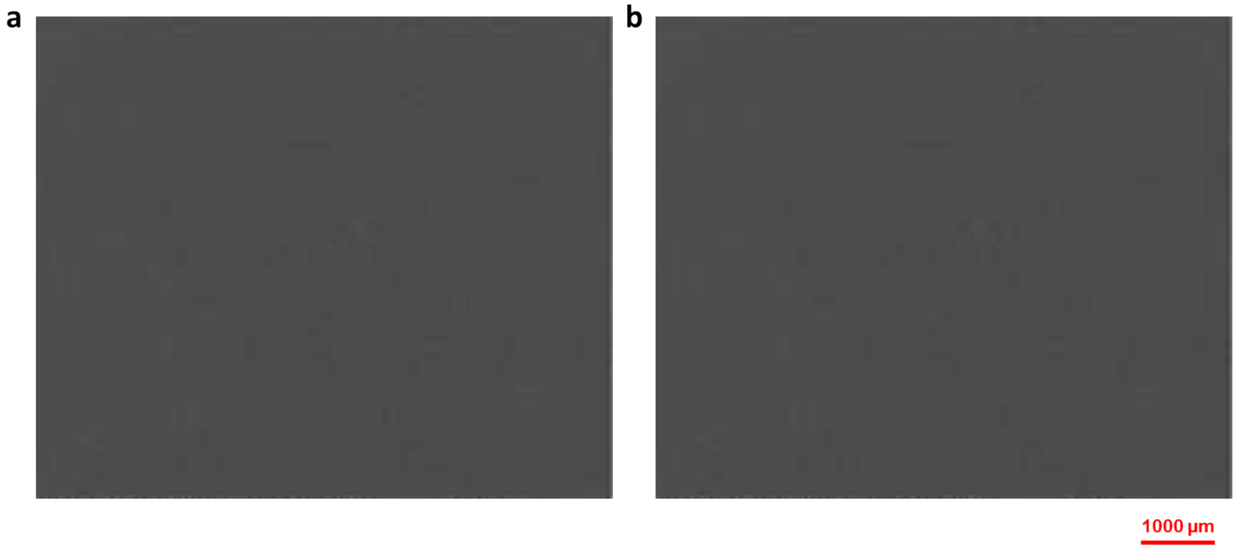

Figure S5: MFNS-CFRP before and after thermal environment adhesion tape testing

a) MFNS-CFRP before the thermal-shock (down to ~ -200˚C) and adhesion tape testing. b) MFNS-CRP after the thermal-shock (down to ~ -200˚C) and adhesion tape testing without sign of deterioration.
